# Compound K, a Ginsenoside Metabolite, Inhibits Colon Cancer Growth via Multiple Pathways Including p53-p21 Interactions

**DOI:** 10.3390/ijms14022980

**Published:** 2013-01-31

**Authors:** Zhiyu Zhang, Guang-Jian Du, Chong-Zhi Wang, Xiao-Dong Wen, Tyler Calway, Zejuan Li, Tong-Chuan He, Wei Du, Marc Bissonnette, Mark W. Musch, Eugene B. Chang, Chun-Su Yuan

**Affiliations:** 1 Tang Center for Herbal Medicine Research, Pritzker School of Medicine, University of Chicago, 5841 S. Maryland Ave., MC 4028, Chicago, IL 60637, USA; E-Mails: zhiyu@uchicago.edu (Z.Z.); gdu@dacc.uchicago.edu (G.-J.D.); cwang@dacc.uchicago.edu (C.-Z.W.); cpuwxd@gmail.com (X.-D.W.); 2 Department of Anesthesia & Critical Care, Pritzker School of Medicine, University of Chicago, 5841 S. Maryland Ave., MC 4028, Chicago, IL 60637, USA; E-Mail: tyler.calway@gmail.com; 3 Section of Hematology/Oncology, Pritzker School of Medicine, University of Chicago, 5841 S. Maryland Ave., MC 4028, Chicago, IL 60637, USA; E-Mail: zjli@uchicago.edu; 4 Department of Orthopaedic Surgery, Pritzker School of Medicine, University of Chicago, 5841 S. Maryland Ave., MC 3079, Chicago, IL 60637, USA; E-Mail: tche@uchicago.edu; 5 Ben May Department for Cancer Research, Pritzker School of Medicine, University of Chicago, 5841 S. Maryland Ave., MC 4028, Chicago, IL 60637, USA; E-Mail: wei@uchicago.edu; 6 Department of Medicine, University of Chicago, 900 E. 57th street, MB 9, Chicago, IL 60637, USA; E-Mails: mbissonn@medicine.bsd.uchicago.edu (M.B.); mmusch@medicine.bsd.uchicago.edu (M.W.M.); echang@medicine.bsd.uchicago.edu (E.B.C.); 7 Committee on Clinical Pharmacology and Pharmacogenomics, Pritzker School of Medicine, University of Chicago, 5841 S. Maryland Ave., MC 4028, Chicago, IL 60637, USA

**Keywords:** colorectal cancer, ginsenoside, compound K, xenograft, cell cycle arrest, p53/p21

## Abstract

Compound K (20-*O*-beta-d-glucopyranosyl-20(*S*)-protopanaxadiol, CK), an intestinal bacterial metabolite of ginseng protopanaxadiol saponins, has been shown to inhibit cell growth in a variety of cancers. However, the mechanisms are not completely understood, especially in colorectal cancer (CRC). A xenograft tumor model was used first to examine the anti-CRC effect of CK *in vivo*. Then, multiple *in vitro* assays were applied to investigate the anticancer effects of CK including antiproliferation, apoptosis and cell cycle distribution. In addition, a qPCR array and western blot analysis were executed to screen and validate the molecules and pathways involved. We observed that CK significantly inhibited the growth of HCT-116 tumors in an athymic nude mouse xenograft model. CK significantly inhibited the proliferation of human CRC cell lines HCT-116, SW-480, and HT-29 in a dose- and time-dependent manner. We also observed that CK induced cell apoptosis and arrested the cell cycle in the G1 phase in HCT-116 cells. The processes were related to the upregulation of p53/p21, FoxO3a-p27/p15 and Smad3, and downregulation of cdc25A, CDK4/6 and cyclin D1/3. The major regulated targets of CK were cyclin dependent inhibitors, including p21, p27, and p15. These results indicate that CK inhibits transcriptional activation of multiple tumor-promoting pathways in CRC, suggesting that CK could be an active compound in the prevention or treatment of CRC.

## 1. Introduction

Colorectal cancer (CRC) is currently one of the most prevalent malignancies in the United States [1,2], making it a major public health concern. Though significant advances have been achieved in early diagnosis and therapy throughout the last few decades, CRC remains the second leading cause of cancer related deaths among American men and the third among American women [3,4]. In fact, the prevalence is rising, yet the five-year survival rate is still poor. Around 50% of patients with advanced disease develop recurrent complications and die soon afterwards [5]. Since numerous CRC patients with advanced disease fail to respond well to current treatment regimens, developing more effective and safer chemopreventive or chemotherapeutic approaches is urgently needed to reduce mortality and obtain better outcomes [6]. In this regard, the evaluation of dietary supplements, which might prevent carcinogenesis and inhibit the growth of CRC, has generated intense interest and achieved some promising success [7,8].

Health benefits derived from plants have been investigated for years. For instance, ginseng has been one of the most popularly consumed traditional medicines for thousands of years in Asian countries such as Korea, Japan and China. Ginseng has been shown to possess diverse pharmacological effects, which appear to be mediated predominantly by saponins, a group of triterpenoid saponins. Some metabolites of ginsenosides transformed by enteric bacteria have shown beneficial activities, including anti-cancer potential [9–11]. Compound K (20-*O*-beta-d-glucopyranosyl-20(*S*)-protopanaxadiol, CK) is the main metabolite of protopanaxadiol-type ginseng saponins synthesized by intestinal bacteria after oral administration of ginseng [12]. CK is believed to be absorbed from the intestine [13,14]. Several reports suggest that CK can inhibit the growth of various tumor cells, such as hepatoma [15,16], lung carcinoma [17], colorectal carcinoma [18,19], and glioma [20] cells by inhibiting cell proliferation and inducing apoptosis. CK has also been shown to enhance the efficacy of anticancer drugs in some drug-resistant cancer cells [21].

It has been shown that over-proliferation and loss of cell cycle regulation are the main factors involved in CRC growth and progression [22]. Some regulators of cell growth, including extracellular signal-regulated kinases (ERKs), cell-cycle regulators and the tumor suppressor gene p53, have been found to be deregulated in the progression of colon cancer [22,23]. Although the anticancer potential of CK has been documented in multiple types of tumor cells, the detailed mechanism, including the effects of CK on cell cycle regulation in CRC cells, has not been examined. In addition, whether CK can inhibit CRC growth *in vivo* has rarely been reported [24].

In the present study, we first observed that CK significantly inhibited tumor growth in a xenograft model of CRC. We also investigated the effects of CK on cell proliferation and apoptosis in human CRC cell lines. Subsequently, we demonstrated that CK arrests the cell cycle at the G1 phase in HCT-116 cancer cells. These observations indicated that CK inhibited cancer cell growth by inducing apoptosis and cell cycle arrest. Our studies suggest that multiple pathways restraining cell growth were upregulated by CK, including transcriptional activation of the ATM/p53-p21 FoxO3a-p27/p15 and TGF-β pathways, which likely contributed to the antitumor effects of CK.

## 2. Results and Discussion

### 2.1. Compound K Inhibits Tumor Growth in a Xenograft Model of Human Colorectal Cancer Cells *in Vivo*

**Figure 1:**
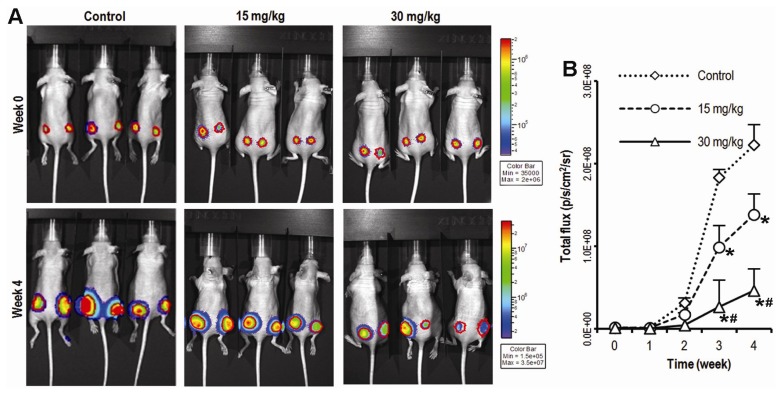
Compound K (CK) inhibits tumor growth in a xenograft model of colon cancer. (**A**) HCT-116 tumor growth was monitored using Xenogen bioluminescence imaging on a weekly basis. Representative Xenogen imaging results at week 0 and week 4 are shown; (**B**) Quantitative analysis of Xenogen bioluminescence imaging. Average tumor sizes at the indicated time points are represented with imaging signal intensities (in photons/second/cm^2^/steradian). (*n* = 5, * *p* < 0.01, compared with control; # *p* < 0.05, compared with the 15 mg/kg group).

Since there has been limited *in vivo* evidence that CK could suppress colon cancer cell growth, we first investigated the anticancer activity of CK using a xenograft model of HCT-116 human colorectal cancer cells. Briefly, exponentially growing firefly luciferase-tagged HCT-116 cells were inoculated into the flanks of athymic nude mice (*n* = 5/group; 1 × 10^6^ cells/site). Beginning on day 1, animals were also administered with CK at 15 or 30 mg/kg (body weight) or vehicle intraperitoneally (IP) every day. Tumor growth was measured by xenogeny bioluminescence imaging on a weekly basis. Representative xenogen imaging results at wks 0–4 are shown in Figure 1A. Quantitative analysis of the imaging data is also presented (Figure 1B). Average tumor size at indicated time points as assessed by imaging signal intensities (in photons/second/cm^2^/steradian) is summarized in Figure 1B. The data showed that the CK treatment group exhibited significantly decreased xenogeny imaging signals compared with the control group. Quantitative analysis revealed that CK significantly inhibited xenograft tumor growth from the 3rd week after CK administration (* *p* < 0.01); the higher dose (30 mg/kg) of CK treatment exhibited a stronger antitumor effect than the lower dose (15 mg/kg) group (# *p* < 0.05), although residual tumors remained. CK, therefore, was significantly capable of suppressing tumor growth *in vivo*.

### 2.2. CK Inhibits HCT-116, SW-480 and HT-29 Cell Viability

**Figure 2:**
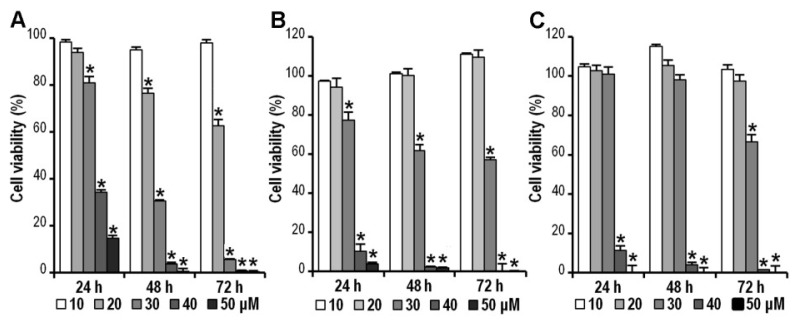
Compound K (CK) inhibits HCT-116, SW-480 and HT-29 colorectal cancer cell viability. Cell survival was determined by MTS assay and calculated as a ratio of the control. CK inhibited HCT-116 cell (**A**), SW-480 cell (**B**), and (**C**) HT-29 cell proliferation in a time- and concentration-dependent manner. Data are presented as mean ± SD from at least triplicate wells and three independent experiments (* *p* < 0.01, compared with control).

To investigate the growth inhibitory effects of CK, human colorectal cancer cell lines HCT-116, SW-480, and HT-29 were treated with different concentrations (10, 20, 30, 40 and 50 μM) of CK for the indicated time (24, 48 or 72 h). Cell viability was assessed by the MTS assay. As shown in Figure 2A–C, the cell viabilities of HCT-116, SW-480, and HT-29 decreased in a dose- and time-dependent manner by doses greater than 20 μM (* *p* < 0.01). HCT-116 cells showed a greater sensitivity to CK treatment at 20 or 30 μM doses than the other two cell lines. HCT-116 expression of the wild-type p53 gene *versus* its deletion or mutation in SW-480 and HT-29 cells, could contribute to the differences between different cell lines. Based on the observation that HCT-116 appeared more sensitive to CK than the other two cell lines, it was selected to investigate anti-cancer mechanisms in the subsequent assays.

### 2.3. CK Promotes Apoptosis in HCT-116 Cells

**Figure 3:**
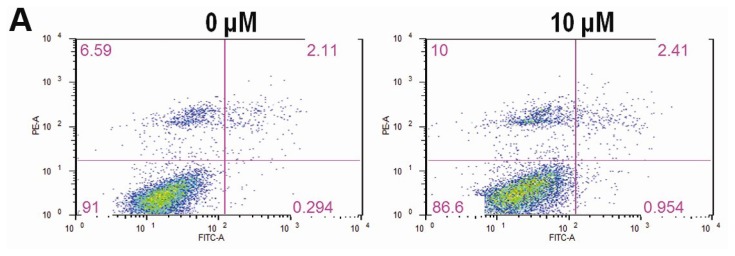

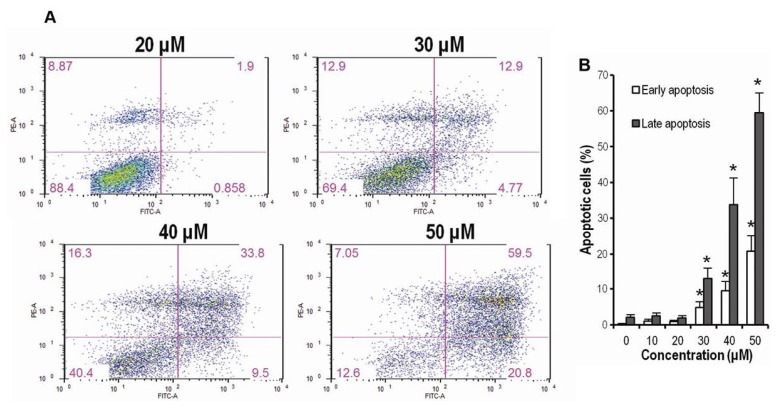
Compound K (CK) promotes early and late apoptosis in HCT-116 cells. (**A**) CK (30–50 μM) induced significant concentration-related apoptosis both early and late in HCT-116 cells compared to the control group; (**B**) Bar plot of CK-induced apoptosis in HCT-116 cells at 72 h (* *p* < 0.01, compared with control). Data are presented as a percentage of total cells counted, 3 dependent assays calculated.

Annexin-V/PI staining assays were employed to investigate whether CK could induce HCT-116 cell apoptosis, since other ginseng extracts have been shown to induce apoptosis in colorectal cancer cells. In this assay, HCT-116 cells were treated with indicated graded concentrations (10–50 μM) of CK for 72 h. Apoptotic cells were determined by flow cytometry using Annexin V/PI double labeling. The fraction of early apoptotic cells (Annexin V-FITC positive) was increased in a dose-dependent manner at doses greater than 30 μM (4.77%, 9.5% and 20.8%, Figure 3A); the fractions of late apoptotic or necrotic cells were also markedly increased compared to DMSO (vehicle) treated cells (12.9%, 33.8% and 59.5%, Figure 3A). Figure 3B showed that CK significantly promoted both early and late stages of apoptosis in HCT-116 cells (* *p* < 0.01) at concentrations greater than 30 μM.

### 2.4. CK Arrests Cell Cycle at the G1 Phase in HCT-116 Cells

**Figure 4:**
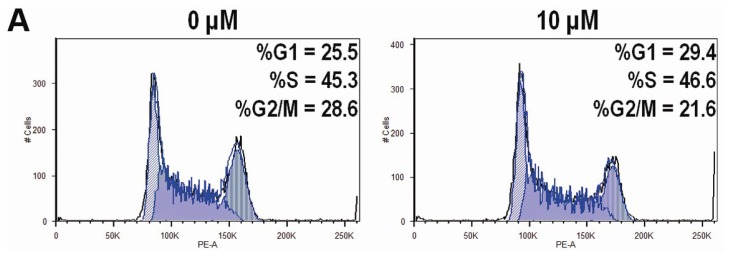

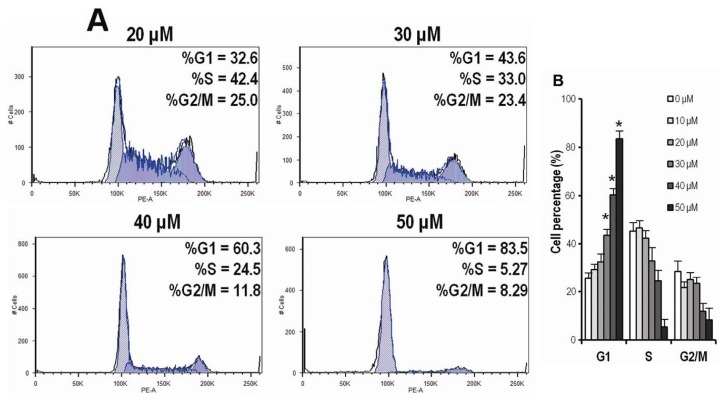
Compound K (CK) induces G1 cell cycle arrest in HCT-116 cells. Data are presented as a fraction of total cells counted. (**A**) CK induces G1 cell cycle arrest in a concentration-related manner; (**B**) Bar plot of CK-induced G1 cell redistribution. * *p* < 0.01, compared with control, 3 dependent assays calculated.

To identify the effect of CK on the cell cycle of HCT-116 cells, we determined the cell cycle distribution of the cells treated with vehicle or different graded concentrations of CK (10–50 μM) for 48 h using PI staining. As shown in Figure 4A,B, CK induced significant G1 cell cycle arrest in a dose-dependent manner at concentrations greater than 30 μM (* *p* < 0.01), with a G1 cell percentage of 43.6%, 60.3%, and 83.5% compared to the control at 25.5%. The FACS data in Figure 4 lacked cells with sub-G1 levels of DNA (thus apoptotic), while data in Figure 3 did show a substantial fraction of apoptotic cells.

### 2.5. CK Induces Gene Expression Changes and Pathways Activation Involved in G1 Cell Cycle Arrest

**Figure 5:**
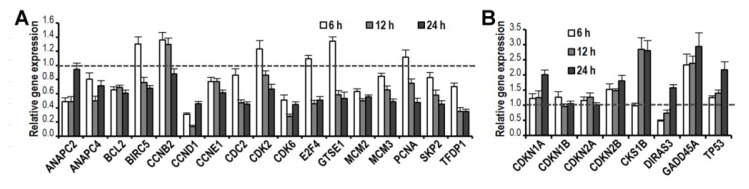

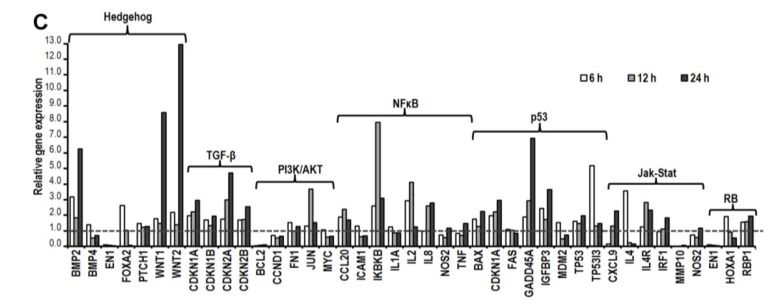
Compound K (CK, 30 μM) promotes changes in several cell cycle-related genes. (**A**) Selected downregulated genes involved in G1 cell cycle transit are shown; (**B**) Selected upregulated genes involved in G1 cell cycle transit are shown; (**C**) CK induces changes in genes related to cell signal transduction. Genes were grouped based on gene ontology categories. Data are presented as mean ± SD; gene expression was normalized using β-actin as a control and calculated by the 2^−ΔΔ^*^c^*^t^ method (*p* < 0.05, compared with control).

Since CK inhibited G1 cell cycle progression, we next examined the effect of CK on the expression of cell cycle-related genes and signaling pathways. We used the RT^2^-profiler PCR array of the human cell cycle and cell signal transduction PCR array plate to test the effects of CK on the target genes in HCT-116 cells. HCT-116 cells were treated with vehicle or 30 μM CK for 6, 12, or 24 h. A large number of cell cycle-related genes were downregulated or upregulated compared to vehicle treated controls. As shown in Figure 5A,B, CK significantly downregulated the expression of several CDKs/cyclins, as well as BCL2, E2F4, GTSE1 and PCNA (*p* < 0.05). In contrast, CK significantly upregulated the expressions of p21^CDKN1A^, p15^CDKN2B^, CKS1B, TP53, GADD45A and DIRAS3 (*p* < 0.05). Most of the genes showed a time-dependent effect within 24 h of CK exposure. In the cell signal transduction pathways array, CK modulated the expression of genes related to important pathways, including the p53, TGF-β, PI3K/AKT, NFκB, and RB at transcriptional level, as shown in Figure 5C.

### 2.6. CK Induces G1 Cell Cycle Arrest in HCT-116 Cells by Activation of Multiple Pathways, Including ATM/p53-p21 and FoxO3a-p27/p15

**Figure 6:**
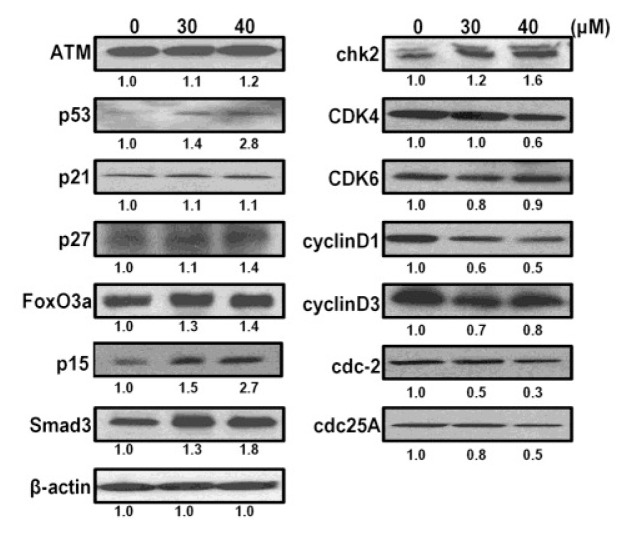
Compound K (CK) induces protein expression related to the G1 cell cycle. Western blots of ATM, p53, p21, p27, p15, CDK4/6-cyclin D1/D3, FOXO3a, Smad3, chk2, cdc2, cdc25A, and β-actin in HCT-116 cells are shown after treatment with CK for 48 h. Band intensity values are presented as a ratio of CK *versus* control and quantified by Image J 1.45 software.

To further clarify the molecular mechanisms induced by CK that drive G1 arrest in HCT-116 cells, we analyzed the protein expression of several potential mediators in lysates from cells treated with vehicle or CK (30 and 40 μM) for 48 h. The results showed that CK increased p53 in a dose-dependent manner in HCT-116 cells (Figure 6). Levels of p21, a cyclin dependent kinase inhibitor, were marginally increased. Ataxia telangiectasia mutated (ATM), which acts as an upstream regulator and co-regulator of p53 in cell cycle processing, was also slightly increased along with p53. It should be noted that p27 and p15 were increased markedly, which suggests that CK-induced G1 cell cycle arrest is not ATM/p53-p21 dependent. CK also increased FoxO3a and Smad3, upstream regulators of p21, p27 and p15. These molecules are the main targets in PI3K/Akt and TGF-β pathway signal transduction. In agreement with the previous PCR array results, this suggests that CK may also drive HCT-116 cell cycle arrest at the G1 phase by modulating the PI3K/Akt and TGF-β pathways. Cdc2 and cdc25A, the effectors of p53/p21, were shown to be downregulated after CK exposure. In brief, the present results suggest that CK induces G1 cell cycle arrest in HCT-116 cells through a complex regulatory network involving multiple pathways, including ATM/p53-p21, PI3K/Akt, and TGF-β, among others.

### 2.7. Discussion

Ginsenosides have been shown to inhibit tumor cell proliferation and tumor growth by inducing differentiation and increasing apoptosis, as well as suppressing tumor cell invasion and metastasis [25–27]. Recent studies reported that CK inhibited human colon cancer cell growth by the induction of apoptosis and cell cycle arrest at the G1 phase through a caspase-dependent pathway via mitochondrial disruption [19,28], or through CAMK-IV/AMPK pathways [18]. In this study, we observed that CK significantly inhibited HCT-116 colon cancer cell growth in an athymic nude mouse model. We also examined the effects of CK on human colorectal cancer cell growth, apoptosis and cell cycle progression, and the possible involvement of signaling pathways including ATM/p53-p21 and PI3K/Akt, TGF-β, *etc*. Based on the findings, we propose that the anti-CRC activity of CK is mainly mediated by these molecular targets. We propose that CK inhibits colorectal tumor cell growth *in vivo* by the induction of G1 cell cycle arrest and promotion of apoptosis.

Previous studies have observed that CK could effectively inhibit growth of a hepatocellular carcinoma cell line, HepG2 [15], making CK a potential candidate for cancer therapy. In addition, other research has shown that CK induced apoptosis in gastric cancer cells via a mitochondrial pathway [29]. Several studies have also reported that CK induced tumor cell arrest in the G0/G1 phase [15,16,30]. Using flow cytometry, we found that CK potently induced HCT-116 cell apoptosis and arrested cells in the G1 phase. However, other researchers have found that CK induced cell cycle arrest in the G2 phase in gastric cancer cells [29], which suggests that CK-induced cell cycle arrest may depend upon the cell context. Our data also suggest that G1 regulators are more sensitive to CK than the regulators of the G2/M phase in HCT-116 cells. As a cell cycle suppressor molecule, the function of p21 is to induce cell cycle arrest by inhibiting CDKs, which has been extensively elucidated before. However, cell cycle regulation is complex and cell-specific. Our results suggest that multiple signal transduction pathways may be involved in CK-induced G1 cell cycle arrest, including FoxO3a, Smad, p15, p27, as well as p21.

The cell cycle is regulated by a series of checkpoints monitoring genomic integrity and ensuring that DNA replication proceeds in a coordinated manner [31]. It is tightly regulated physiologically by CDK inhibitors (CKIs). The progression of the cell cycle is regulated by the balance of CDKs and CKIs through a network of growth-inhibitory and growth-stimulatory transduction signals [32,33]. D-type cyclins have been shown to be unstable and are degraded mainly via the 26S proteasome in a ubiquitin-dependent manner [34]. A number of therapeutic agents have been observed to induce cyclin D1 degradation [35,36]. These studies indicate that the induction of cyclin D1 degradation may offer a useful approach for therapeutic intervention against cancer. Based on our findings, CK could induce CKI upregulation and downregulate cyclin D expression, both of which could contribute to G1 arrest and support the potential of CK in colorectal cancer treatment. Since cell proliferation and differentiation are specifically controlled in the G1 phase and the G1–S transition in the cell cycle, oncogenic progresses exert the greatest effect by targeting particular regulators of G1 phase progression [37]. Any chemical or biological agent that could induce G1 phase cell cycle arrest would play a pivotal role in controlling cell differentiation and progression during the carcinogenesis process. As we showed in this study, CK induced G1 phase cell cycle arrest and apoptosis in human colorectal carcinoma cells, which indicated that CK could be an important agent for colorectal cancer chemoprevention and chemotherapy.

The tumor suppressor gene p53 is a key mediator in the induction of cell cycle arrest and apoptosis following DNA damage or cellular stress in human cells [38,39]. Although p53 itself is not a CKI, it has been implicated in the induction of G1 phase cell cycle arrest and apoptosis through transactivation of a number of downstream genes [40,41]. Among the transcriptional targets of p53, p21 plays a key role in mediating G1 arrest [42]. We observed that CK induced G1 phase arrest in HCT-116 cells, which possess functional p53, and increased p53 and p21 protein levels. This indicated that these two genes are the major targets of CK. Two of the major mechanisms involve inhibitory binding of p21 to the CDK via the p53/p21 pathway and an increase in inhibitory phosphotyrosine levels of CDK [43–45]. A previous study demonstrated that the p53/p21 pathway is critical for chemotherapy-induced G1 phase arrest [46]. Since p53 can inhibit the cell cycle and induce apoptosis, pharmacological strategies to control or restore wild-type p53 function could have great therapeutic potential. Cell cycle arrest that is dependent on p53 requires transactivation of p21 or other cell cycle-related factors [33].

A previous study showed that inactivation of CDK4/6 in a human mammary epithelial cell line was the result of the ability of TGF-β to repress expression of the CDK tyrosine phosphatase cdc25A [47]. In our investigation, it was evident that treatment with CK in HCT-116 cells increased the expression of p15 in a dose-dependent manner, whereas the expression of p21 was not increased notably, suggesting that the expression of p15 may play a more important role in G1 phase arrest than p21. In addition, the increase in FoxO3a and p27 and the downregulation of cdc25A may both contribute to the inhibition of CDK/cyclin activity during this form of differentiation. CK also significantly increased levels of Smad3, a key intracellular mediator of TGF-β, which suggested that the TGF-β pathway is also involved in the G1 arrest induced by CK. Consistent with the signal pathway PCR arrays, it should be noted that multiple pathways including p53/p21, PI3K/Akt, TGF-β, and RB, among others, were involved in cell phenotype variations, such as changes in apoptosis or the cell cycle after exposure to CK.

## 3. Experimental Section

### 3.1. Chemicals and Reagents

Ginsenoside compound K (CK) was obtained from Chromadex (Irvine, CA, USA), diluted to 10, 20, 30, 40 and 50 mM in DMSO (Fisher Chemicals, Fair Lawn, NJ, USA), and stored in small aliquots for later use at −20 °C.

### 3.2. Cell Culture Conditions

Human colorectal cancer cell lines HCT-116, HT-29, and SW480 were obtained from the American Type Tissue Collection (Rockville, MD, USA) and maintained in McCoy’s 5A or L-15 medium (Hyclone, Logan, UT, USA). HCT-116-Luc cell line that stably expresses firefly luciferase was generated and manipulated as described previously [48,49]. All media were supplemented with 10% fetal bovine serum (FBS), penicillin (100 IU/mL), and streptomycin (100 μg/mL). The cells were trypsinized twice a week and incubated at 37 °C, 95% humidity, 5% CO_2_.

### 3.3. Xenograft Tumor Model of Human Colon Cancer, Xenogen Bioluminescence Imaging

Female athymic nude mice (4–6 weeks of age, Harlan, Indianapolis, IN, USA) were used. The use and care of animals was carried out under the guidelines approved by the Institutional Animal Care and Use Committee. Subconfluent HCT-116-Luc cells were harvested and resuspended in PBS to a density of 2 × 10^7^ cells/mL. Prior to inoculation, cell viability was tested by 0.4% trypan blue exclusion assay (viable cells > 90%). For subcutaneous injection, approximately 1 × 10^6^ HCT-116-Luc cells in 50 μL PBS were injected into both flanks of each mouse for each point. Starting the same day, CK was intraperitoneally (IP) administered at doses of 15 or 30 mg/kg (body weight) every day. Control mice were injected with vehicle. Animal whole body optical imaging was carried out as described previously [50]. Animals were subjected to Xenogen IVIS 200 imaging system (Caliper Life Sciences, Hopkinton, MA, USA) for imaging weekly after tumor cell inoculation. d-luciferin sodium salt (Gold Biotechnology, St. Louis, MO, USA) at 100 mg/kg body weight in 0.1 mL sterile PBS was administered IP as a substrate before imaging. Acquired pseudo images were collected by superimposing the emitted light over the grayscale photographs of the animal. Quantitative image analysis was performed with Xenogen’s Living Image V2.6.1 software.

### 3.4. MTS Assays

HCT-116, SW-480, and HT-29 cells were seeded in 96-well plates at a concentration of 5000 cells/well, allowed to attach overnight and then exposed to different concentrations of CK (10, 20, 30, 40 and 50 μM). Cell proliferation was measured at 24, 48, and 72 h later using the Cell Titer 96 Aqueous MTS Reagent (Promega, Madison, WI, USA) according to the manufacturer’s protocol. The absorbance was measured by an automated microplate reader (Epoch; Bio-Tek Instruments, Winooski, VT, USA) at 490 nm. Results were expressed as a ratio of treated cells *versus* control (vehicle set at 100%).

### 3.5. Apoptosis Assay

5 × 10^4^ HCT-116 cells were seeded in 24-well plates. After 24 h, CK was added at the indicated concentrations. After 72 h treatment, all adherent (trypsin digest) and non-adherent floating cells were collected, and centrifuged for 5 min at 600*g*. Then the cells were stained with Annexin-V (FITC) and propidium iodide (PI) (Becton Dickinson, San Diego, CA, USA) according to the manufacturer’s instructions. The stained cells were subsequently analyzed by a FACS Canto flow cytometer (Becton Dickinson, Mountain View, CA, USA). All experiments were performed in three independent plates, and run in triplicate each time. At least 10,000 cells were counted each time for analysis.

### 3.6. Cell Cycle Assay

For cell cycle analysis, 1 × 10^5^ HCT-116 cells were seeded in 12-well plates. On the second day, CK or vehicle was added. Treated cells were cultured for 48 h, and then all adherent cells were collected by trypsin, fixed with 80% ethanol and stored for 2 h at −20 °C. After treatment with 0.25% Triton X-100 for 5 min, the cells were resuspended in 50 μL of PI/RNase staining buffer (Becton Dickinson, San Diego, CA, USA), incubated in the dark for 20 min at room temperature, and counted with a FACS Canto flow cytometer. At least 10,000 cells were counted for each measurement.

### 3.7. Real-Time PCR Array for Cell Cycle and Signal Pathway Analysis

Cells were treated with 30 μM CK or vehicle for the indicated times (6, 12, or 24 h) and then total RNA was extracted using an RNeasy mini kit (Qiagen, Valencia, CA, USA) and quantified by Nanodrop (Thermo, Wilmington, DE, USA). The first strand of cDNA was prepared using a RT^2^ first strand kit (SABioscience, Frederick, MD, USA). Then, the transcriptional product was analyzed using real-time PCR analysis. A human cell cycle RT^2^ Profiler PCR array plate (Cat# PAHS-020E, 84 genes covered, SABioscience, Frederick, MD, USA) and a Human Signal Transduction PathwayFinder PCR array plate (Cat# PAHS-014E, 84 key genes covered, SAbioscience, Frederick, MD, USA) were used for screening following the manufacturer’s instructions. Experiments were repeated three times. Relative gene expression levels were determined using the 2^−ΔΔ^*^c^*^t^ method.

### 3.8. Immunoblot Assay

HCT-116 cells were collected 48 h after exposure to 30 or 40 μM CK or vehicle and then lysed in cold radio immunoprecipitation assay (RIPA) buffer supplemented with 1% (*v*/*v*) protease inhibitor cocktail, centrifuged, and stored in aliquots at −80 °C for later analysis. The protein concentration of the samples was determined by a BCA protein assay kit (Pierce, Rockford, IL, USA). Lysates (40 μg protein) were denatured by heating at 95 °C for 10 min and loaded on 4%–15% Mini-PROTEAN TGX precast gels (Bio-Rad, Hercules, CA, USA) for electrophoresis. Then, the proteins were transferred to a PVDF membrane (Bio-Rad, Hercules, CA, USA) and blocked in PBST buffer (PBS with 0.05% Tween 20) containing 5% nonfat dried milk. The blots were incubated with the indicated primary antibodies (p53, p21^waf1/cip1^, p27^Kip1^, p15^INK4B^, ATM, FoxO3a, Chk2, Smad3, CDK4/6, cyclin D1/3, cdc2, cdc25A and β-actin from Cell Signaling, Danvers, MA, USA) overnight at 4 °C, followed by incubation for 1 h with the appropriate secondary antibodies conjugated with horseradish peroxidase in blocking buffer. β-actin was measured as the loading control. Immunocomplexes were detected using SuperSignal West Pico Substrate (Thermo-pierce, Rockford, IL, USA) following the manufacturer’s directions, and quantified by densitometry using Image J software. Intensities were normalized to vehicle-treated samples after adjusting for β-actin loading.

### 3.9. Statistical Analysis

Data are expressed as mean ± SE or SD as indicated. Comparisons of two groups were made using student’s *t*-test. A *p* value set at 0.05 was used to determine significant differences. All analyses were performed using SPSS 14.0 (IBM Corporation, Somers, NY, USA).

## 4. Conclusions

In summary, the present study demonstrates that: (a) human colorectal cancer cells are highly sensitive to growth inhibition by CK; (b) reduced survival of HCT-116 cells after exposure to CK is associated with G1 cell cycle arrest and induction of apoptosis; (c) CK can inhibit cell cycle progression at the G1 phase by increasing expression of p53, p27, p15, FoxO3a and Smad3, and by decreasing the expression of cdc2, cdc25A, CDK4, CDK6, cyclinD1, and cyclin D3; and (d) CK-induced cell growth inhibition in HCT-116 cells is mediated by multiple pathways, including ATM/p53-p21, PI3K/Akt, and TGF-β, among others, that induce G1 cell cycle arrest and apoptosis. In addition, an *in vivo* assay showed that CK potently inhibited growth of HCT-116 tumor xenografts in a dose-dependent manner. These findings suggest that CK may be a promising agent to prevent colonic tumorigenesis or even treat established CRC in combination with other agents.
